# Does physical activity level have an impact on long-term treatment response in temporomandibular disorders: protocol for a prospective study

**DOI:** 10.1186/s12903-022-02428-3

**Published:** 2022-09-14

**Authors:** Youngwoo Chun, Jung Hwan Jo, Ji Woon Park

**Affiliations:** 1 Department of Oral Medicine, Seoul National University Dental Hospital, 101, Daehak-ro, Jongno-gu, Seoul, 03080 Republic of Korea; 2 Department of Oral Medicine and Oral Diagnosis, School of Dentistry, Seoul National University, 101 Daehak-ro, Jongno-gu, Seoul, 03080 Republic of Korea; 3 Dental Research Institute, Seoul National University, 101 Daehak-ro, Jongno-gu, Seoul, 03080 Republic of Korea

**Keywords:** Temporomandibular disorders, Physical activity, Accelerometry, Exercise, Inflammation, Comorbidity

## Abstract

**Background:**

Temporomandibular disorders (TMD) is a disease characterized by pain and dysfunction of the masticatory muscles and temporomandibular joint. Many factors have been found to be related to the disease however, the underlying mechanism is yet to be fully elucidated. Physical activity is widely known to modulate pain intensity in various pain disorders. However, literature suggesting the association between physical activity and signs and symptoms of TMD are limited.

**Methods and design:**

The “Physical Activity in TMD (PAT)” is a prospective study on TMD patients that aims to determine how daily physical activity and sleep duration affect long-term TMD prognosis following conventional treatment. To analyze such effects, objective data on daily physical activity levels will be collected along with clinical indices including mouth opening ranges and masticatory muscle palpation responses from adult Koreans diagnosed with TMD following standardized diagnostic procedures. Well-known comorbidities of TMD will be extensively evaluated based on validated structured questionnaires on sleep quality, fatigue level, widespread pain, psychological status including depression and anxiety, autonomic symptoms, and health-related quality of life. The collected data will be analyzed according to TMD pain severity and physical activity level, and correlations among physical activity indices and long-term TMD prognosis will be investigated.

**Discussion:**

In this longitudinal prospective study of adult Koreans diagnosed with TMD following standardized diagnostic procedures, primary outcomes include physical activity levels and long-term TMD clinical outcomes and secondary outcomes include disability from pain and related comorbidity levels. Results and analysis are ongoing. The results of this study will provide reliable data for future research and establish clinical guidelines that will allow cause-related, patient-tailored personalized medicine for TMD.

*Trial registration*: Clinical Research Information Service (Registration number: KCT0007107). Registered March 22 2022 https://cris.nih.go.kr/cris/search/detailSearch.do?search_lang=E&focus=reset_12&search_page=M&pageSize=10&page=undefined&seq=21420&status=5&seq_group=21420.

## Background

Temporomandibular disorders (TMD) is a disease usually represented by discomfort and/or dysfunction of the masticatory muscles and temporomandibular joints (TMJ) leading to impairment of basic daily functions such as chewing and speaking. It is known as the most prevalent cause of nonodontogenic pain in the orofacial region, which 5–12% of the adult population experience [1]. Many factors have been identified to cause and exacerbate its symptoms including sleep quantity and quality, genetic, hormonal, anatomical, and psychosocial factors [2, 3].

Physical activity has been known to affect symptoms in various pain disorders. Exercise is significantly associated with lower perceived pain levels in fibromyalgia patients, while high-intensity exercise was reported to increase pain levels in fibromyalgia patients [4]. Low back pain is also known to be aggravated by higher activity levels [5]. The underlying mechanisms of such interactions are not fully understood. The increase in inflammatory mediators with high-intensity exercise may directly cause an increase in pain levels [6]. However, another previous study reported lower inflammatory biomarker levels including high sensitivity C-reactive protein (hsCRP) with moderate to vigorous physical activity [7], suggesting a complicated interaction between the two. Contradictory results can also be found in clinical reports regarding pain disorders, which involve regular physical activity [8, 9]. Such inconsistencies are largely due to the heterogeneity of the studies in defining physical activity levels and patient groups.

In spite of its profound impact on quality of life and high prevalence, no previous study has prospectively investigated the correlation between TMD and physical activity while considering well-known confounders such as psychological status and sleep quality in addition to hematological markers of systemic inflammation. Since the quantity and quality of sleep can significantly affect pain, it is also necessary to control its influences. Physical activity, sleep duration, and TMD pain are strongly interrelated as they affect one another through overlapping mechanisms.

## Objectives

Therefore, the main objectives of this “Physical Activity in TMD (PAT)” study are,

1. To assess the long-term effect of objective physical activity level and sleep duration on clinical characteristics in well-defined TMD patient groups.
2. To analyze the impact of physical activity on treatment response to conventional TMD management approaches by determining the cut-off value for physical activity that affects treatment prognosis.
3. To define the causal relationship between abnormal physical activity levels and sleep durations on chronic, high-intensity TMD pain, its comorbidities, and systemic inflammatory marker levels.

## Methods and design

### Participants, interventions, and outcomes

#### Study design

This is a longitudinal cohort study being conducted prospectively in Seoul National University Dental Hospital from May 2021. It was approved by the Institutional Review Board (IRB) of the same hospital (#CRI21007). The study is registered in Clinical Research Information Service, an online registration system for clinical researches (registration number KCT0007107). The system includes all items from the trial registration data set of the World Health Organization (version 1.3.1). Written informed consent is collected from all participants by YC. Further modifications to the study protocol that occur after IRB approval will be reported to the review board and relevant parties for additional permission. In case of any major modification of protocol version 1.0, the issue will be thoroughly discussed with the IRB. Whenever there is a change, a new protocol version will be assigned. All procedures complied with multiple ethical standards of the institutional research committee, the Helsinki declaration in 1964, and its later amendments or other equivalent ethical standards.

#### Participants and eligibility criteria

Adult patients who are 18 years old or older with Korean nationality are included as participants. They visited the outpatient clinic of the Department of Oral Medicine of Seoul National University Dental Hospital with the chief complaint of pain and dysfunction of the temporomandibular joint and were diagnosed with TMD. The diagnoses were implemented according to the Diagnostic Criteria for TMD (DC/TMD) regardless of TMD subtype diagnosis or following treatment plan to allow further subgroup analysis [10]. Patients with previously diagnosed systemic musculoskeletal disorders including fibromyalgia, low back pain, rheumatoid arthritis, ankylosing spondylitis, gout, liver disease, kidney disease, an uncontrolled endocrine disorder, autoimmune disease, trauma within the last 6 months, treatment history for a psychiatric disorder that may affect the study, and primary sleep disorder diagnosis will be excluded.

#### Interventions

All participants were treated with identical protocols after TMD diagnosis. The treatment protocols include conservative treatment such as consultation (explanation of the etiology and anatomy of the temporomandibular joint), management of contributing factors including tooth clenching, physical therapy (moist hot pack, ultrasound therapy, low-level laser therapy, medication, and transcutaneous electrical nerve stimulation), and occlusal splints with full coverage of the upper teeth with hard resin.

To promote the retention of participants, staff will contact those that are lost at the designated follow-up without rescheduling. Data from those that withdraw their consent to participate will not be included in the final analysis.

#### Recruitment of cases

Data will be gathered sequentially from all participants visiting the participating hospital for TMD and provide written informed consent from July 2021. Recruitment will proceed for 12 months and may be prolonged when the number of cases is insufficient. The final sample size is estimated to be approximately 200 participants based on a previous study on rheumatoid arthritis using statistical sample size estimation methods (α = 0.05, β = 0.10) [11].

#### Patient grouping

TMD patients diagnosed with DC/TMD are grouped according to two different criteria.

##### Pain disability level evaluated with graded chronic pain scale (GCPS) of DC/TMD axis II

1. *Low disability TMD group* GCPS grade I and II (low disability-low intensity pain and low disability-high intensity pain, respectively).
2. *High disability TMD group* GCPS grade III and IV (high disability-moderately limiting pain and high disability-severely limiting pain, respectively)

##### Amount of time counted as moderate to vigorous physical activity (MVPA)

1. *Inactive* less than 150 min per week engaging in moderate-intensity physical activity and less than 75 min per week engaging in vigorous-intensity physical activity
2. *Active* 150 min or more per week engaging in moderate-intensity physical activity or 75 min or more per week engaging in vigorous-intensity physical activity

#### Study outcomes

##### 3.1.6.1. Primary outcome measures: clinical outcomes listed below will be gathered at the participant’s first visit (baseline) and at 6- and 12-month post-treatment.

**Fig. 1:**
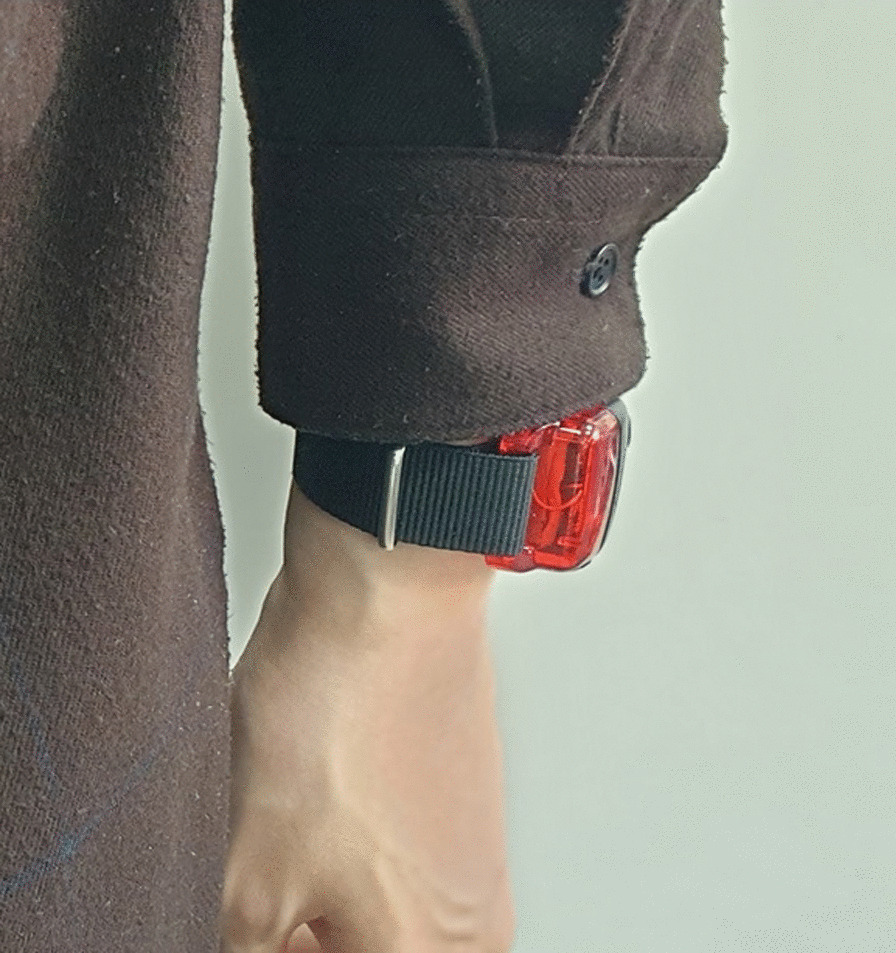
Wrist worn accelerometer

- Amount of physical activity and sleep duration objectively measured using ActiGraph wGT3X-BT (ActiGraph, Florida, USA), a validated 3-axis accelerometer that includes ambient light sensors. Actigraphy is a method to monitor the cycles of rest and activity, which does not involve any invasive procedures. The movements of the accelerometer and light exposure can be continually recorded with a watch-like device worn on the wrist to objectively determine the amount of physical activity and sleep duration of a participant. Despite its limitations in measuring and staging sleep, actigraphy data provides real-life information on both the objective amount of physical activity and sleep by allowing the participant to maintain normal routines in his or her natural environment. Physical activity is measured at baseline only. (Fig. 1)
- *Amount of comfortable mouth opening* [12] the participant will be asked to open his or her mouth as wide as possible while remaining pain-free. If the participant is already experiencing pain, open as wide as possible without increasing current pain. Interincisal distance between the maxillary and mandibular reference teeth will be measured.
- *Amount of maximum mouth opening* [12] the participant will be asked to open as wide as possible, even if it is painful. Interincisal distance between the maxillary and mandibular reference teeth will be measured.
- *Muscle tenderness on palpation* [12] 12 masticatory muscle areas will be palpated on both sides including posterior, middle, and inferior temporalis muscle area and origin, body, and insertion of the masseter muscle with 1 kg of pressure.
- *Joint capsule tenderness on palpation* [12] 6 areas of TMJ will be palpated on both sides including the posterior, superior, and lateral parts of the capsule with 0.5 kg of pressure in a passive state. When palpation elicits subjective pain, the response will be recorded as positive. Joint noises are verified through palpation.
- *Subjective pain intensity* participant reported pain intensity based on a 0–10 numeric rating scale (NRS).

##### Secondary outcomes: comorbidity and systemic inflammation levels

- Series of validated structured questionnaires to assess comorbidity levels (Table 1)
- Hematologic indices of systemic inflammation: red blood cell (RBC) levels showed significant correlations with pain intensity before treatment, comfortable mouth opening, pain on palpation of cervical muscles, and mouth opening range at 6 months after treatment [33]; hsCRP and erythrocyte sedimentation rate (ESR) are non-specific markers of inflammatory activity used in patients of rheumatoid arthritis or spondyloarthritis [34]; platelet-to-lymphocyte ratio (PLR), neutrophil–lymphocyte ratio (NLR), and derived neutrophil–lymphocyte ratio (dNLR) are widely used inflammatory biomarkers showing significant correlation with disease activity and mortality [35–38].

**Table 1:** List of structured questionnaires to measure comorbidities

Section	Questionnaire	Description
Physical activity	International physical activity questionnaire (IPAQ) [13]	assess physical activity level based on self-report
	Tampa scale of kinesiophobia for temporomandibular disorders (TSK-TMD) [14, 15]	assess the severity of exaggerated, incoherent, and debilitating fear of movement and activity
Sleep disturbance and fatigue	Pittsburgh sleep quality index (PSQI) [16]	assess the quality of sleep over a one-month period
	Epworth sleepiness scale (ESS) [17]	assess the daytime sleepiness of the patients
	Fatigue assessment instrument (FAI) [18]	assess fatigue and distinguish normal fatigue from fatigue-related medical disorders
	Insomnia severity index (ISI) [19]	assess the severity of insomnia
	Morningness−eveningness questionnaire (MEQ) [20]	investigate morningness and eveningness of patients as to when the subject would prefer to start sleep or wake up, rather than when he or she actually does
Widespread pain	Symptom severity (SS) scale [21]	diagnose fibromyalgia in patients based on the adapted 2010 American College of Rheumatology fibromyalgia survey criteria. And also, to assess the severity of widespread body pain and centralized pain characteristics
	Widespread pain index (WPI) [21]	
	Fibromyalgia impact questionnaire (FIQ) [22]	measure status, progress and outcomes of fibromyalgia-like widespread pain by assessing physical performance, work condition, depression, anxiety, tiredness in the morning, pain, rigidity, fatigue, and well-being over the one-week period
Psychologic disturbance	Symptom checklist-90-revised (SCL-90-R) [23]	evaluate psychological problems and psychotic symptoms
	Beck depression index (BDI) [24]	evaluate presence of pathological levels of depression over the recent one-week period
	Beck anxiety index (BAI) [25]	assess the severity of physical and cognitive symptoms originating in anxiety over the recent one-week period
	Pain catastrophizing scale (PCS) [26]	assess the patient’s tendency to exaggerate the threat of a pain stimulus and to feel helpless
	Central sensitization inventory (CSI) [27]	identify patients who have symptoms that may be related to central sensitization or central sensitivity syndromes
	Pennebaker Index of Limbic Languidness (PILL) [28]	measure an individual’s tendency to notice an array of physical symptoms and sensations
	Perceived stress scale (PSS) [29]	measure nonspecific perceived stress
General health	Short form 36 (SF-36) [30]	evaluate difficulties in various activities including physical, social, and usual role, bodily pain, mental health in general, vitality, and general perceptions of health
	Composite autonomic symptom score 31 (COMPASS 31) [31]	score autonomic symptom severity in domains including orthostatic intolerance, vasomotor, secretomotor, gastrointestinal, bladder, and pupillomotor
	Short form McGill pain questionnaire (MPQ) [32]	document the quality and intensity of pain experienced by the patient

**Fig. 2:**
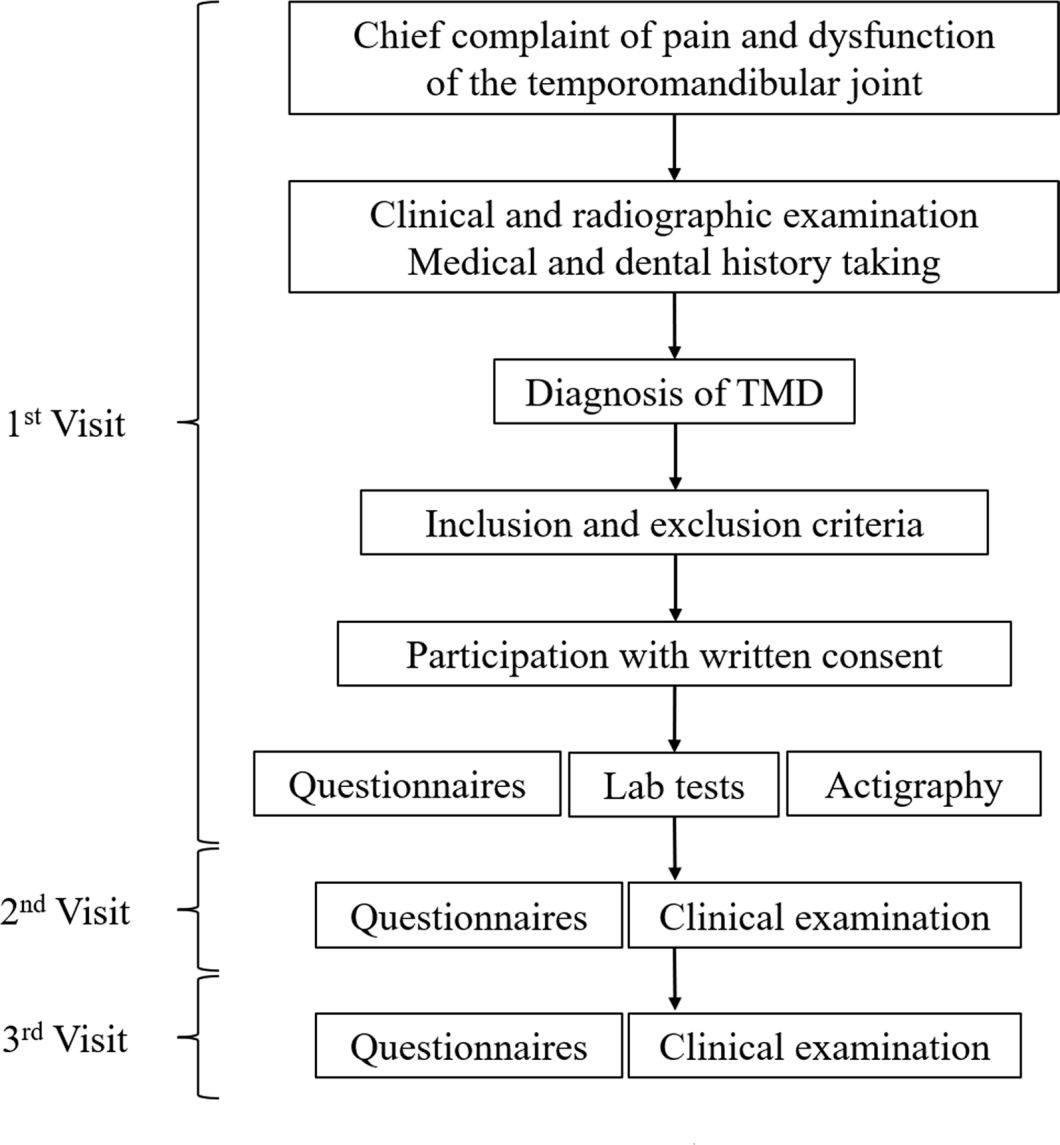
Flowchart of study process

The study flow is given in Fig. 2.

We hypothesized that physical activity level could influence the long-term prognosis of pain and clinical indices of TMD in addition to sleep duration and quality, and other comorbidities related to psychosocial aspects.

### Data management and availability

The medical record number of each participant will be gathered to search for the required data.

Clinical and surveyed information of each participant will be recorded on a secure server and paper print-outs. Then the necessary information will be documented in an Excel file, which can be utilized for statistical analyses. The participants will be assigned a consecutive number and the names will not be disclosed in compliance with the International Conference of Harmonization for Good Clinical Practice recommendations [39]. The fact that their obtained information will be confidentially stored in a computer with high security will be notified to all participants. The participants’ written consent will be stored by the principal investigator. All data will be confirmed and authenticated by a biostatistician at Seoul National University. Since this study is an observational study rather than a clinical trial study, a data monitoring committee is not required. The principal investigator is in charge of any interim analyses and cessation.

The data gathered in this study will not be able to be accessed publicly owing to ethical issues however, will be available upon reasonable request from the corresponding author.

### Sample size calculation

Sample size estimation for this study was based on the primary outcome of physical activity level measured in minutes and 77.8 min as between-group difference. A total of 214 participants (107 per group) will be needed based on analysis to reach the power of 90% with an alpha error of 0.05 and an estimated study dropout rate of 20%.

### Statistical analysis

The obtained data will be analyzed using SPSS Statistics ver. 26.0 software (IBM Co., Armonk, NY, USA). The significance level will be set to 0.05. To compare differences between groups, both parametric and nonparametric tests will be adopted accordingly. Descriptive statistics for clinical variables and comorbidity levels will be presented as mean with standard deviation, and median with lower and upper quartiles. Logistic regression will be used to investigate factors associated with a worse long-term prognosis of TMD in terms of pain and function. All potential confounders, including clinical indices of TMD symptoms measured before treatment, body mass index, blood pressure and pulse rate, scores reflecting psychosocial status, sleep quality, and hematologic indices will be included as independent variables along with actigraphy data such as the amount of energy consumption per day, metabolic equivalent of task, steps count, sleep time, wake after sleep onset, sleep efficiency, sleep fragment index, and movement index. Factors will be added first in order to make adjustments, after which will be discarded consecutively one at a time with the first being the one with the highest *p*-value. The cutoff point of confounders to be discarded will be set as *p* < 0.250. If the *p*-value is 0.250 ≤ *p* ≤ 0.300, the confounder will not be discarded if this affects the OR by more than 10% [40]. Receiver operating characteristic (ROC) curves will be analyzed to explore thresholds for potential biomarkers. Their prognostic values will also be assessed by using Kaplan–Meier curve and both univariate and multivariate COX regression models. If the estimated sample size is not reached, multiple imputation will be used for missing data. A single overall analysis result will be produced by combining multiple analysis results.

## Discussion

More accurate data collection is possible when actigraphy is applied because activity data is objectively measured rather than depending on participant self-reporting. Also, unlike certain questionnaires such as the Godin–Shephard leisure-time exercise questionnaire (GSLTPAQ) [41], which only ask about leisure time, actigraphy measures the amount of activity for 24 h regardless of the activity type. For measuring sleep, compared to polysomnography (PSG) actigraphy has the great advantage that it can measure sleep time during the day. Furthermore, the influence of selection bias is also reduced because compliance is high, especially when it is worn on the wrist [42–44]. Unlike PSG, actigraphy is relatively easier to be done on consecutive days. A longer measurement period can significantly improve validity [45]. Therefore, wrist-worn accelerometers are being used in large population surveys such as the UK BioBank study, the Whitehall II cohort, and the US National Health and Nutrition Examination Survey (NHANES) [46–49]. In spite of such advantages, actigraphy has some limitations. Actigraphy does not provide information on eye movements (EOG), brain activity (EEG), heart rhythm (ECG), or muscle activity (EMG). To overcome such shortcomings, a certain amount of recording time is required for each sleep indices. For sleep percent, at least 2 nights are required, 5 nights for sleep efficiency, and 7 nights for total sleep time when using only actigraphy [50]. Although actigraphy shows less accuracy, it has been reported that it is possible to measure sleep parameters relatively accurately without a sleep diary. Actigraphy may even show a higher association with some sleep disorders including circadian rhythm disorders and insomnia compared to PSG [51]. With the development of technology, it is expected that the accuracy of actigraphy measurements will be further improved in the future.

Physical activity has a bidirectional relationship with sleep [52]. Moderate-level exercise is recommended as a non-pharmacological treatment for sleep problems. It was reported that patients with chronic insomnia took 7 min less to fall asleep and had 17 min longer total sleep time after 4 weeks of regular exercise [53]. In a study of 6 months of exercise, total sleep time increased by 27 min, and sleep efficiency also increased by 11% [54]. In respect of subjective sleep quality, middle-aged and older-aged adults who have sleep problems were reported to show moderate improvements with exercise training in a meta-analysis of 6 studies [55]. Conversely, sleep problems can also affect physical activity. Adults who complained of poor sleep were less active than those with better sleep. For example, compared with adults without insomnia, those with insomnia symptoms are less active and have lower cardiorespiratory fitness, and this may be due to daytime sleepiness and/or fatigue [56–58]. In addition, adults with sleep-disordered breathing are more likely to be physically inactive than those without sleep-disordered breathing, which shows that sleep-disordered breathing severity is reversely correlated with objective indices of physical activity [59, 60]. Such subsided activity levels frequently result from excessive weight, insufficient energy, and elevated levels of fatigue and sleepiness, which are typical aspects of adults with sleep-disordered breathing [61, 62].

The association between physical activity and mental health has also been continuously investigated. A recent systematic review summarized that depressive patients had reduced daily activity and lengthened wake after sleep onset, and the treatment of depression improved not only wake after sleep onset but also sleep efficacy and latency [63].

The current literature generally supports the fact that higher physical activity level is associated with lower systemic inflammation. CRP levels were more associated with the frequency of daily MVPA than the total accumulated time of weekly MVPA [64]. According to the National Health and Nutrition Examination Survey (NHANES), CRP levels were reversely associated with the amount of time spent engaging in leisure-time physical activity [65]. However, in British men, CRP levels were not significantly related to the amount of physical activity level [66]. It was concluded that the duration of physical activity does not seem to matter, and MVPA was considered to be more important. In a study using actigraphy, sedentary time was positively associated with greater CRP levels [67]. A randomized control trial compared CRP levels of participants physically active and inactive, and results showed lower CRP levels in the active group [68]. Also, CRP levels were positively correlated with longer sitting times. Such relations could also be found in populations with other diseases [69]. When it comes to individuals with arthritis, ≤ 150 min per day of MVPA or ≥ 4000 of daily step counts could be associated with lower hsCRP levels [70]. In fibromyalgia patients, 10–35 min of MVPA per day and 5000–9000 daily steps were associated with lower hsCRP levels [70].

The accumulating evidence supports the intimate relationship between physical activity, sleep quality, and systemic inflammation which are all factors known to influence TMD symptoms in previous literature [71–74]. Through this prospective study based on objective physical activity measurements in a well-defined patient group of TMD, the resulting data will be able to support the establishment of a clinical guideline related to physical activity recommendations and the diagnostic value of such measurements in TMD prognosis.

## Trial status

The protocol is version 1.2 (June 24, 2021). The first recruitment began on July 21, 2021. Recruitment period ended on May 5, 2022. The participants will be followed for 12 months after their first intervention and this will be approximately February 25, 2023.

Data collection is in process. The trial submission was postponed due to delays in trial registration caused by the COVID-19 pandemic related working issues.

## Data Availability

The datasets generated during and/or analysed during the current study are not publicly available due to ethical reasons but are available from the corresponding author on reasonable request.

## Abbreviations

ANA: Antibody to antinuclear factor
anti-CCP: Anticyclic citrullinated peptide antibody
BAI: Beck anxiety index
BDI: Beck depression index
CI: Confidence intervals
COMPASS 31: Composite autonomic symptom score 31
CRP: C-reactive protein
CSI: Central sensitization inventory
DC/TMD: Diagnostic criteria for temporomandibular disorders
dNLR: Derived neutrophil lymphocyte ratio
ECG: Electrocardiogram
EEG: Electroencephalogram
EMG: Electromyography
EOG: Electrooculogram
ESR: Erythrocyte sedimentation rate
ESS: Epworth sleepiness scale
FAI: Fatigue assessment instrument
FIQ: Fibromyalgia impact questionnaire
GCPS: Graded chronic pain scale
GSLTPAQ: Godin–Shephard leisure-time exercise questionnaire
hsCRP: High sensitivity C-reactive protein
IPAQ: International physical activity questionnaire
ISI: Insomnia severity index
MEQ: Morningness–eveningness questionnaire
MET: Metabolic equivalent of task
MPQ: McGill pain questionnaire
MVPA: Moderate to vigorous physical activity
NHANES: National Health and Nutrition Examination Survey
NLR: Neutrophil lymphocyte ratio
OR: Odds ratios
PAT: Physical activity in TMD
PCS: Pain catastrophizing scale
PILL: Pennebaker index of limbic languidness
PLR: Platelet-to-lymphocyte ratio
PSG: Polysomnography
PSQI: Pittsburgh sleep quality index
PSS: Perceived stress scale
RBC: Red blood cell
RF: Rheumatoid factor
ROC: Receiver operating characteristic
SCL-90-R: Symptom checklist-90-revised
SF-36: Short form 36
SS: Symptom severity
TMD: Temporomandibular disorder
TMJ: Temporomandibular joint
TSK-TMD: Tampa Scale of Kinesiophobia for Temporomandibular Disorders
VM: Vector magnitude
WPI: Widespread pain index
